# School engagement profiles in Chilean secondary students

**DOI:** 10.3389/fpsyg.2022.1088089

**Published:** 2023-01-25

**Authors:** Ximena de Toro, Mahia Saracostti, Laura Lara, Horacio Miranda, Edgardo Miranda-Zapata

**Affiliations:** ^1^Universidad Autónoma de Chile, Beca de Doctorado Nacional ANID, Santiago, Chile; ^2^Cátedra UNESCO Bienestar de la Niñez y Juventud, Educación y Sociedad, Escuela de Trabajo Social, Universidad de Valparaíso, Valparaíso, Chile; ^3^Núcleo Científico Tecnológico en Ciencias Sociales y Humanidades, Universidad de la Frontera, Temuco, Chile; ^4^Departamento de Psicología, Universidad Autónoma de Chile, Santiago, Chile; ^5^Departamento de Psicología Evolutiva y de la Educación de la Universidad de Sevilla, Sevilla, Spain; ^6^Departamento de Producción Agropecuaria, Universidad de La Frontera, Temuco, Chile; ^7^Facultad de Educación, Universidad Autónoma de Chile, Temuco, Chile; ^8^Centro de Investigación Escolar y Desarrollo (Cied-UCT), Universidad Católica de Temuco, Temuco, Chile

**Keywords:** school engagement, profiles, academic achievement, family, peers, teachers, gender, secondary school

## Abstract

School engagement is considered a key variable in promoting educational trajectories. Previous research shows that maintaining high levels of school engagement is fundamental, given its association with multiple academic results and lower-risk behaviors. This article aims to show how school engagement profiles (based on the behavioral, affective, and cognitive subdimensions) relate to academic achievement (math and language), contextual factors (family, teachers, and peer support), and gender. This study involved 527 students enrolled in the 1st year of secondary education in public schools in Chile. All students came from vulnerable schools. Our study used cluster analysis to identify students’ profiles. We identified the existence of three different profiles of school engagement (high, medium, and low) considering the three subdimensions of school engagement (behavioral, affective and cognitive). Secondly, ANOVA analysis showed differences in language and math academic achievement scores between the profiles, where higher engagement students showed higher academic performance in language and math. These findings are consistent with previous studies showing that contextual factors strongly influence school engagement and better behavioral engagement in female than male students. It will discuss the pertinence of person-centered approaches focusing on combinations of variables within students rather than taking each variable as the focal point when analyzing goals. These techniques are a favorable methodological alternative to investigate why some students have better results than others instead of just ranking students by their performance. It will conclude with some future lines of research and practical implications.

## Introduction

School Engagement (SE) is defined as the participation of the student in their educational process (Saracostti et al., 2019). SE is a multidimensional construct composed of affective, cognitive, and behavioral dimensions (Lara et al., 2021). Lara et al. (2018) and Saracostti et al. (2019, 2022) define affective engagement as the emotional response towards the learning process and the school, characterized by feeling part of a school community and involved. Behavioral engagement is related to participation in academic activities, attendance, and social interactions, whether at home, in face-to-face or online school. Thirdly, the cognitive dimension is linked to the awareness and willingness to build learning, the interest in using different deep learning strategies, and the effort to develop learning skills.

The measurement of SE has been a reliable indicator of the degree to which students participate in daily educational activities, being important to maintain SE levels as high as possible, given its association with multiple academic outcomes and academic performance (Lei et al., 2018; Wang et al., 2019; Chambers and Michelson, 2020; Canet-Juric et al., 2021).

When students are engaged, they see learning as meaningful, are motivated, and show commitment to their learning and future. SE is considered a key variable in the promotion of educational trajectories. This variable gained more attention than attendance after the pandemic of COVID 19 when being online was not the most effective proxy to assess which students were on and off track with their studies. After the pandemic, schools need a better measure of school accountability. Additionally, SE goes beyond attendance, task completion, or compliance with adult directives. When students are engaged, they feel a sense of belonging and safety and experience success. Thus, they want to attend school (Miranda-Zapata et al., 2018). Moreover, it is essential to maintain levels of SE as high as possible, given its association with multiple academic results and lower-risk behaviors. School disengagement relates to the initiation of substance use, conduct problems, violence, school dropout, and delinquency (Li et al., 2011; Lam et al., 2014; Høigaard et al., 2015; Lei et al., 2018; Canet-Juric et al., 2021). Contrarily SE is a protective factor contributing to academic and overall success since it is linked to students’ positive mental health, well-being outcomes, and healthy behaviors (Griffiths et al., 2012; Fredricks et al., 2019). Thus, it may mitigate the pandemic and post-pandemic stress among students, Thus, it may contribute to mitigate pandemic and post-pandemic stress among students.

SE is influenced by contextual factors (*CF*), and that greater knowledge about these will help to develop interventions in favor of CE. Among those factors on which the school can intervene, unlike the structural factors that are little modifiable in the short and medium term, are: (1) family support; (2) peer support, and (3) teacher support (Lara et al., 2021; Navarro et al., 2021; Sotomayor et al., 2021). Family support is the perception of help in the learning process in case of problems and the motivation and interest families provide to students. Peer support refers to students’ perceptions of interpersonal interactions, concern, and trust with their peers. Finally, the third factor refers to the perceived support and motivation to learn provided by the teaching team in academic aspects and when the students have personal problems. Since external factors influence SE, current approaches conceive it as a highly malleable state (Fernández-Zabala et al., 2016; Ansong et al., 2017; Saracostti et al., 2019; Lara et al., 2021; Navarro et al., 2021).

These *CF* make sense from the ecological model (Bronfenbrenner and Morris, 2006) for which the dynamics and relationships that occur in and between the most immediate systems of children, such as family and school, have a significant impact on their development. Consequently, social support from family, peers, and teachers stand out as the main factors related to SE to prevent school dropout and contribute to the adjustment of adolescent students and positive educational trajectories (Fredricks et al., 2016; Fernández-Lasarte et al., 2019; Lara et al., 2021; Miranda-Zapata et al., 2021; Saracostti et al., 2021).

This conceptualization of SE and *CF* does not place the focus exclusively on the characteristics and abilities of students, focusing on them all the responsibility for their educational process; to also encompass those family and school contextual elements that contribute decisively to their configuration (Fredricks et al., 2004). In this sense, the context would become essential in building engagement. This conception of SE as a malleable process is highly relevant, opening the possibility of implementing interventions and changes that schools may promote (Lam et al., 2014; Fredricks et al., 2016). In this way, schools can offer a more optimistic alternative to achieve positive results by focusing on those variables on which they can exert some effect (Appleton et al., 2006).

In the case of Chile, the studies on the subject revealed the mediating role played by the SE concerning the influence of *CF* on students’ academic performance and attendance (Miranda-Zapata et al., 2018, 2021). Thus, the measurement of SE and *CF* can provide relevant clues to schools to timely observe these variables and make informed and contextualized decisions. Although in Ibero-America, the concern for SE and the need to have diagnostic, monitoring, and evaluation systems have been installed progressively, progress toward intervention strategies in this area is still very incipient ( De-Toro et al., 2021). Unlike Anglo-Saxon countries (Vansteenkiste et al., 2009; Xie et al., 2020), where there are studies to identify profiles of engaged students considering behavioral, affective, and cognitive dimensions, in Chile and Ibero-America there is no research that allow us to identify which profiles are associated with these variables. This information could guide students better and support the decision at the public policy and school level (Fredricks et al., 2019).

Additionally, researchers (Fredricks et al., 2004; Lam et al., 2014; Wilcox et al., 2018) have examined gender differences in SE and academic performance in school. Results reported that girls usually have higher levels of SE. These studies highlight the importance of understanding the multiple factors influencing engagement, including gender, to provide targeted promotion and intervention strategies.

To advance in this area, researchers (Fredricks et al., 2019) suggest person-centered analytic techniques that allow us to examine “the possibility that students may not be uniformly engaged across all dimensions, but rather, that there are subgroups of students characterized by unique clusters of engagement” (Bae et al., 2020, p: 2). Thus, focusing on the combinations of variables rather than taking a single one as the focal point may be helpful since this kind of analysis can be used to develop programs that are relevant to the needs of students who may present different engagement or disengagement profiles (Fredricks et al., 2019). According to such interest, this article aims to analyze how different school engagement profiles (based on the behavioral, affective, and cognitive components) relate to academic achievement (math and language), contextual factors (family, teachers, and peers), and sex through non-parametric analysis techniques such as unsupervised neural network clusters belonging to machine learning.

## Materials and methods

### Participants

This study involved 527 students enrolled in the 1st year of secondary education from 11 public schools, aged between 14.2 and 17.6 years (M = 15.3, SD = 0.68), 54.1% being female students (*n* = 285) and 45.9% male students (*n* = 242). According to the School Vulnerability Index calculated annually by the National School Aid and Scholarship Board, all students came from schools with a high level of school vulnerability. This index took into consideration family socioeconomic context, access to the health system, housing quality, and parents’ educational level, among other factors (Saracostti et al., 2022).

### Instruments

School Engagement Questionnaire: a self-report instrument composed of 29 items that measure three subdimensions of SE: affective (10 items, e.g., “I feel that the school cares about me”), cognitive (12 items, e.g., “for me it is important to understand the assignments and subjects well”), and behavioral [7 items, e.g., “I leave the classroom without asking permission (or I leave the online classes)”], with a response scale from 1 (never or almost never) to 5 (always or almost always). In this article, we use the version adapted and validated for the context of virtual, face-to-face, or hybrid classes in the Chilean educational system caused by the Covid-19 pandemic (Lara et al., 2022), which maintains the same number of items as the same content and presents the same factorial structure of three correlated factors as the original version (Lara et al., 2018). The results of the confirmatory factor analysis showed a good fit of the data to the three-factor (affective, behavioral, and cognitive) correlated model (χ^2^ = 964.314; df = 374; RMSEA = 0.052/CI = 0.048–0.056; CFI = 0.925; TLI = 0.919) as well as reliability analyses showed adequate internal consistency of the entire scale (α = 0.907) and its component dimensions (α = 0.859 for affective dimension, α = 0.718 for behavioral dimension and α = 0.885 for cognitive dimension).

Contextual Factors Questionnaire: an instrument composed of 18 items that measure the three primary *CF* that influence SE: family [3 items, e.g., “. I talk to my family about what I do at school (or in online classes)”], teachers (8 items, e.g., “I get along with my teachers”), and peers (7 items, e.g., “My classmates support me and care about me”), with a response scale from 1 (never or almost never) to 5 (always or almost always). In this article, we use the version adapted and validated for the context of remote, face-to-face, or hybrid classes in the Chilean education system caused by covid-19 (Lara et al., 2022), which maintains the same number of items with the same content and presents the same factorial structure of three correlated factors as the original version (Navarro et al., 2021). The results of the confirmatory factor analysis showed a good fit of the data to the three-factor (family, teacher and peer support) correlated model (χ^2^ = 414.047; df = 132; RMSEA = 0.061/CI = 0.054–0.067; CFI = 0.963; TLI = 0.957) as well as reliability analyses showed adequate internal consistency of the entire scale (α = 0.917) and its component dimensions (α = 0.753 for family dimension, α = 0.881 for teacher dimension and α = 0.898 for peer dimension).

Academic performance in math and language: Students’ academic achievement was evaluated through the final average grade obtained at the end of the school year in math and language, recorded on a scale from 1 (minimum achievement) to 7 (maximum achievement). It will consider maths and language since most research agrees on interpreting academic performance through achievements in mathematics and language, highlighting the importance of both areas for involvement in society. Mathematical thinking train children in strategies that are essential in everyday life, more with the increase in technology and digitization. While language and communication are the other leading tools to understand the world, relate to others, be part of a cultural community, and develop critical and reflective thinking (Archambault et al., 2009; Graves and Brown Wright, 2011; Boonk et al., 2018; Fung et al., 2018; Masrai and Milton, 2018; Pace et al., 2019).

### Procedures

This study is part of a more comprehensive research project titled “Modeling of school engagement, social, family and school contextual factors and socio-educational trajectories of children and adolescents: from the international scientific literature to a longitudinal study in the Chilean context” (ANID/FONDECYT 1210172). Previous research results are available.^1^

This is a longitudinal study focused on investigating and analyzing the meanings that children and adolescents in Chilean schools construct about a predictive model of trajectories of SE (cognitive, behavioral, and affective subdimension), *CF* (family, peers and teachers support) and socio-educational achievements (academic performance, permanence, approval of subjects, attendance, others) over time. Differences according to gender and educational cycle (primary and high schools) have been explored.

The sampling method was non-probabilistic expert judgment, selecting schools and students with similar characteristics. The participating schools were contacted through the leading researcher or the research team and invited to participate voluntarily. All the contacted schools decided to participate in the study. The data collection was carried out virtually and collectively during school hours. In this way, members of the research team duly trained for this purpose made the necessary explanations and answered the students’ questions through the virtual platforms they used to attend their virtual classes. Once the questionnaires were explained, the students completed them through an online platform developed for this purpose ( De-Toro et al., 2021; Saracostti et al., 2022), with an approximate duration between 30 and 40 min.

This study has the approval of the ethics committees of the Universidad de Valparaíso. In order to participate in this study, the participants had to sign an informed assent and have the signed informed consent of their legal representatives.

### Data analysis

The statistical analyses were applied to variables with different minimum and maximum ranges, since they corresponded to the sum of different numbers of items with Likert-type responses of 1 to 6 points, which also did not have a normal distribution, so nonparametric methods were selected, such as the Kohonen (1982) unsupervised neural network cluster (SOM), and the number of clusters was validated using the Bayesian information criterion (BIC). To compare the clusters, the Kruskal Wallis test and the Dwass-Steel-Critchlow-Fligner (DSCF) *post-hoc* test were applied, which corrects the alpha error of multiple pairwise comparisons.

## Results

### School engagement profiles

**Figure 1:**
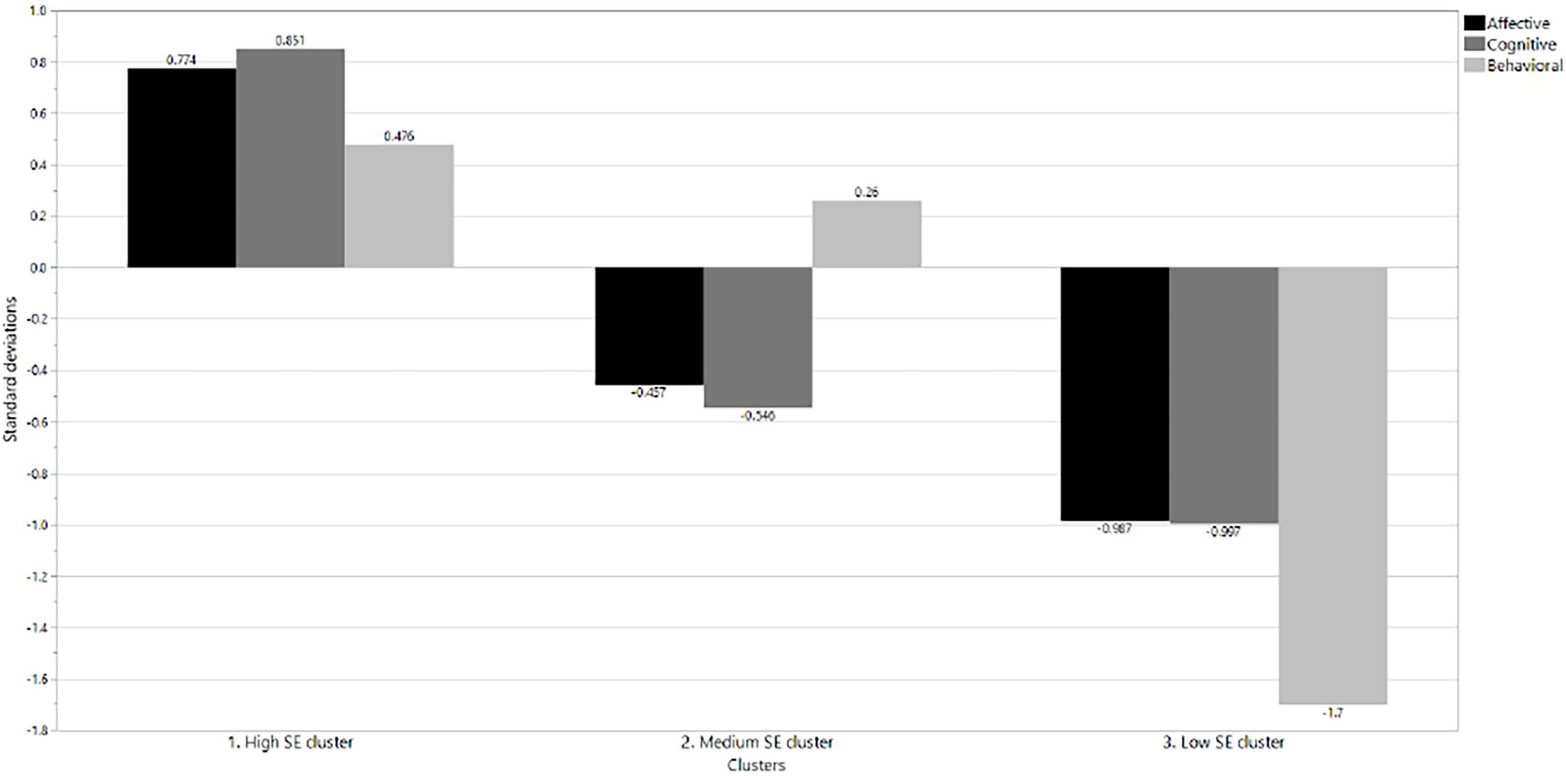
School engagement profiles.

Cluster analyzes showed that optimal solution was grouping the participants into three clusters, which were named according to the School Engagement level, in: High-level, Medium and Low SE (Figure 1). The number of clusters was evaluated by assessing the variability within and between clusters and considering the comparative results for discussion purposes with the results of other publications on this subject.

Since the distribution of the profiles was found not to approximate the normal distribution, researchers calculated a nonparametric one-way Kruskal-Wallis ANOVA and the effect size η2 on the standardized scores of the three subscales of SE with the clusters as comparison factors. The Kruskal-Wallis and Welch’s comparative tests were evaluated and presented the same conclusions of statistical significance (*p* ≤ 0.01), which allowed us to conclude that variability did not generate differences in the conclusions of the tests.

The results showed that all the subscales differed significantly depending on the cluster, presenting an effect size greater than 0.14, considered large (Cohen, 1988) in all the comparisons of the SE subscales: affective engagement: χ^2^(2) = 289, *p* < 0.001, η^2^ = 0.549; cognitive engagement: χ^2^(2) = 339, *p* < 0.001, η^2^ = 0.645; behavioral engagement: χ^2^(2) = 242, *p* < 0.001, η^2^ = 0.460. Descriptive statistics are presented in Table 1.

**Table 1:** Descriptive statistics for dimensions of SE by levels.

	High SE mean (SD)	Medium SE mean (SD)	Low SE mean (SD)
SE affective	4.32 (0.41)	3.30 (0.68)	2.86 (0.69)
SE cognitive	4.23 (0.42)	3.12 (0.58)	2.77 (0.53)
SE behavioral	4.75 (0.31)	4.62 (0.33)	3.45 (0.47)

The analysis of the effect of clusters on the family, teacher and peer scales was considered through the statistical significance analysis of clusters pairs comparison tests.

The differences post-hoc test Dwass-Steel-Critchlow-Fligner pairwise comparisons (DSCF; Steel, 1960) of the SE and *CF* between the clusters are presented in Table 2.

**Table 2:** Comparison of the sums of the SE and *CF* scales between pairs of cluster levels.

	High – medium		High – low		Medium – low	
	W	*p*-W	W	*p*-W	W	*p*-W
Affective	20.53	0.001	18.24	0.001	7.54	0.001
Cognitive	22.75	0.001	19.16	0.001	18.78	0.001
Behavioral	7.34	0.001	19.92	0.001	18.78	0.001
Family	14.31	0.001	14.74	0.001	3.82	0.001
Teachers	15.56	0.001	17.89	0.001	10.85	0.001
Peers	11.90	0.001	11.35	0.001	1.02	0.751

The comparison of the affective, cognitive, and behavioral factors showed a total differentiation between the clusters according to the SE, with statistically significant differences *p* ≤ 0.05 in all the factors.

Based on these differences and scores, the first cluster was labeled high-level SE, representing 237 students (45.0%) who scored above the mean on all subscales (0.476 to 0.851 standard deviations). The second cluster was called medium SE, including 194 students (36.8%) with scores around the mean on the school engagement scales (from −0.546 to 0.260 standard deviations). The third cluster was low SE, including 96 students (18.2%) who scored below average on all school engagement subscales (de −0.987 a − 1.7 standard deviations).

### Differences among SE profiles

The results of the Kruskal-Wallis ANOVA between the clusters according to levels of SE showed that there were highly significant differences (*p* ≤ 0.001) in all the variables studied (family, teachers and peer support, as well as in academic performance in mathematics and language), although with different effect sizes: family support factor [χ^2^(2) = 155.8, *p* < 0.001, η^2^ = 0.296]; teacher’s support factor [χ^2^(2) = 228.5, *p* < 0.001, η^2^ = 0.434]; peers [χ^2^(2) = 98.8, *p* < 0.001, η^2^ = 0.188]; academic performance in language [χ^2^(2) = 53.2, *p* < 0.001, η^2^ = 0.101]; and in mathematics [χ^2^(2) = 48.1, *p* < 0.001, η^2^ = 0.091].

As can be seen in Table 3, concerning the distribution of *CF*, it is observed that the high-level SE cluster scored higher in the family support factor (M = 4.16, SD = 0.88) than the ones in the middle SE cluster (M = 3.12, SD = 1.05), and the low SE cluster (M = 2.78, SD = 0.84).

**Table 3:** Descriptive statistics for contextual factors, academic performance and sex by SE levels.

	High SE mean (SD)	Medium SE Mean (SD)	Low SE mean (SD)
Contextual factors			
Family	4.16 (0.88)	3.12 (1.05)	2.78 (0.84)
Teachers	4.43 (0.50)	3.61 (0.77)	2.82 (0.74)
Peers	3.54 (0.96)	2.67 (1.01)	2.54 (0.86)
Academic performance			
Language	5.79 (0.76)	5.44 (0.86)	5.13 (0.72)
Mathematics	5.96 (0.75)	5.63 (0.80)	5.32 (0.77)
Sex	*n* (%)	*n* (%)	*n* (%)
Female students	141 (59.49)	103 (53.09)	41 (42.71)
Male students	96 (40.51)	91 (46.91)	55 (57.29)

In addition, it is observed that the cluster with high-level SE scored higher in the teacher’s support factor (M = 4.43, SD = 0.5) than ones in the middle SE cluster (M = 3.61, SD = 0.77), and the low (M = 2.82, SD = 0.74). The results are similar in relation to achievement in mathematics. Also, high-level SE cluster scored higher in the peer support factor (M = 3.54, SD = 0.96) than the ones in the middle SE cluster (M = 2.67, SD = 1.01) and the low (M = 2.54, SD = 0.86).

Regarding academic performance, the high-level SE cluster shows greater achievement in language (M = 5.79, SD = 0.76) than the middle SE cluster (M = 5.44, SD = 0.86), and the low (M = 5.13, SD = 0.72). In addition, the high-level SE cluster shows a higher achievement in mathematics (M = 5.96, SD = 0.75) than in the middle SE cluster (M = 5.63, SD = 0.8) and the low (M = 5.32, SD = 0.77).

Concerning the distribution of the proportion based on sex, the high-level and middle SE clusters have a higher proportion of female students than male students but do not show significant differences between them [χ^2^(1) = 1.779, *p* = 0.182]. On the other hand, the low SE cluster has a higher proportion of adolescents and significant differences with the high-level SE cluster [χ^2^(1) = 7.767, *p* = 0.005] but not with the middle SE cluster [χ^2^(1) = 2.770, *p* = 0.096].

## Discussion

From the results presented, we can conclude the existence of three different profiles of SE (high-level, medium, and low) considering the main subdimension of SE (cognitive, affective, and behavioral). Secondly, results showed differences in language and math academic achievement scores between the SE profiles, where high-level SE students showed higher academic performance in language and math. These findings are consistent with previous research (Lei et al., 2018), supporting the importance of school engagement for academic achievement. Similarly, Yuwen and Yanping (2021) discuss the relationship between school engagement and the learning effect in virtual classes, showing associations between more behavioral engagement and the learning effect; specifically, their analysis shows that the more behavioral engagement, the more positive impact on the learning effect.

Additionally, results also showed differences in the support students received from their families, peers, and teachers. In this way, our results showed that high-level SE students reported more support from these three contexts. These findings align with previous studies highlighting the importance of family, peers, and teachers as the critical contexts for SE (Winter, 2013; Fernández-Zabala et al., 2016; Miranda-Zapata et al., 2018; Navarro et al., 2021). For example, the high-level SE cluster scored higher in the family factor, teachers, and peers than in the middle and low SE. Consistently, Roundfield et al. (2018) show that *CF* and, in particular, peer support affect the three dimensions of SE, while the family mainly affects adolescents’ behavioral and cognitive engagement. In addition, Quin et al. (2018) highlight that family, teachers and peers strongly influence some academic and emotional indicators of SE. Fernández-Zabala et al. (2016) identified significant correlation rates between *CF* (family support, teachers support, and peers support) and SE. Finally, Kiefer et al. (2015) concluded that teachers’ and peers’ support positively affects the academic motivation of adolescents, SE, and school belonging. In addition, they found significant relationships between teachers’ support (autonomy, structure, and participation), peer support (academic and emotional), and adjustment (motivation, commitment, and belonging).

These results show that person-centered techniques, such as cluster or profile analysis, are a favorable methodological alternative to investigate why some students have better results than others, instead of just ranking students by their performance (Bulgarelli et al., 2020). A person-centered approach focuses on particular combinations of motivational variables within individuals or groups of students rather than taking each variable as the focal point (Regueiro et al., 2018) when analyzing goals. Thus, research findings of person-centered analysis can enhance the development of more personalized strategies and provide teachers with more tools to improve educational trajectories.

Another scope of this study is to include the gender perspective, considering that the absence of the gender perspective in educational research may lead to inaccurate conclusions and suggestions for both school leaders and educational policymakers (Ovseiko et al., 2016). Regarding gender differences, our results show that there are more girls with high-level of SE and more male students with low SE, with no differences regarding the medium cluster. These findings are consistent with previous studies that show that girls were more behaviorally engaged than male students (Havik and Westergård, 2020).

In the same vein, Wilcox et al. (2018) examine the relationship between gender and SE and found that for male students, grade level is significantly associated with SE, but it is not a significant variable for girls, while for female students, anxiety is significantly associated with SE but it is not a significant variable for male students. They concluded that considering the social and relational nature of SE, a focus on contextual factors highlighted in this article, and a profound understanding of the differences between boys’ and girls’ engagement, is essential to develop effective interventions and improve practices and outcomes for students.

On the other hand, studies (Fredricks et al., 2019) show that SE decreases as one moves on to secondary education. Thus, it is interesting to carry out longitudinal studies to investigate how SE profiles change throughout the educational trajectory. In addition to that, more research is needed to examine how these profiles relate to academic adjustment and achievement in higher education. Studies in this area (van Rooij et al., 2017) revealed that behavioral and cognitive subdimensions are related to better adjustment and performance in higher education.

Fredricks et al. (2019) also identified from a literature review three profiles of students with low school engagement (1) behaviorally disengaged, (2) emotionally disengaged, and (3) cognitively and emotionally disengaged students. These results provides clues that not only students with behavioral problems are more likely to present risk behaviors in their educational trajectories, but also those students with emotional problems with their schools. Thus, future lines of research should look more closely at those with low school engagement to help schools comprehensively identify and reflect on why students are losing school engagement and design more relevant interventions.

Other promising lines link SE profiles and achievement in specific areas, such as sciences (Bae et al., 2020), which would be interesting to observe in the country based on the gender variable, taking into account the existing gaps in the participation of women in science and research. Finally, in order to enhance the identification of guidelines for the intervention and improvement of educational processes future research should consider other *CF* such as educational practice and school support services.

Among the study’s limitations, we must point out the intentionality of the sample. In this sense, the data obtained has the bias of coming only from contexts of high social vulnerability, which is why it would be necessary to analyze whether profiles change in more diverse contexts. In addition, the data was taken during a pandemic, when a group of students was no longer participating in virtual classes due to the consequences that the closure of schools brought to education at an international level. This socio-sanitary crisis also implied costs to families, in peer support, and in the same teachers worried about their students and their own families (Kuric Kardelis et al., 2021; OECD, 2021). Therefore, the challenge remains to investigate how these profiles behave over time now that face-to-face classes have resumed.

As shown, the degree of SE and *CF* is a critical element in students’ academic achievements, especially during secondary school, highlighting the need to generate scientific evidence from the Chilean context. Some practical implications of this study are that the measures of SE and *CF* (Lara et al., 2022) allow taking decisions about strategies to promote school engagement according the different profiles. Results from our study corroborate that SE can contribute, as in other cultural contexts, to implement policies to promote it. These scientifically validated indicators may give schools clues about what decisions to make to improve students’ educational trajectories (Bravo-Sanzana et al., 2021). However, collecting data on these indicators to prevent school dropouts is not enough. It is required to have robust technical components connected to a network of interventions in order to detect, but also help students who need support in their educational trajectories (De-Toro et al., 2021).

Future lines of research could investigate the implications of these different profiles in other patterns of development processes and psychological adjustment, along with looking more deeply at the *CF.* It is necessary to consider the different profiles and not just study students’ goals, as good behavior or performance, independently. For instance, Engels et al. (2019) identified how status profiles were related to their peer group and their relationship with SE, with the most popular students having the lowest levels of behavioral engagement.

## Data availability statement

The datasets generated and analysed during the current study are not publicly available due the fact that they constitute an excerpt of research in progress but are available from the corresponding author on reasonable request.

## Ethics statement

The studies involving human participants were reviewed and approved by Ethics Committee of the Universidad de Valparaiso and the Chilean National Commission for Scientific and Technological Research. Written informed consent to participate in this study was provided by the participants’ legal guardian/next of kin.

## Author contributions

XT, MS, and LL drafted the manuscript, contributed to the interpretation of the data, and provided important critical revisions. XT, MS, and EM-Z developed the study concept, the study design, and arrange the data collection. HM, LL, and EM-Z made the statistical analysis. All authors contributed to the article and approved the submitted version.

## Funding

This work was supported by ANID/FONDECYT 1210172.

## Conflict of interest

The authors declare that the research was conducted in the absence of any commercial or financial relationships that could be construed as a potential conflict of interest.

## Publisher’s note

All claims expressed in this article are solely those of the authors and do not necessarily represent those of their affiliated organizations, or those of the publisher, the editors and the reviewers. Any product that may be evaluated in this article, or claim that may be made by its manufacturer, is not guaranteed or endorsed by the publisher.

## Footnotes

^1^ https://compromisoescolar.com/recursos/material-de-estudios
